# A fast radiotherapy paradigm for anal cancer with volumetric modulated arc therapy (VMAT)

**DOI:** 10.1186/1748-717X-4-48

**Published:** 2009-10-25

**Authors:** Florian Stieler, Dirk Wolff, Frank Lohr, Volker Steil, Yasser Abo-Madyan, Friedlieb Lorenz, Frederik Wenz, Sabine Mai

**Affiliations:** 1 Department of Radiation Oncology, University Medical Center Mannheim, University Heidelberg, Germany; 2 Department of Radiation Oncology and Nuclear Medicine (NEMROCK), Faculty of Medicine, Cairo University, Egypt

## Abstract

**Background/Purpose:**

Radiotherapy (RT) volumes for anal cancer are large and of moderate complexity when organs at risk (OAR) such as testis, small bowel and bladder are at least partially to be shielded. Volumetric intensity modulated arc therapy (VMAT) might provide OAR-shielding comparable to step-and-shoot intensity modulated radiotherapy (IMRT) for this tumor entity with better treatment efficiency.

**Materials and methods:**

Based on treatment planning CTs of 8 patients, we compared dose distributions, comformality index (CI), homogeneity index (HI), number of monitor units (MU) and treatment time (TTT) for plans generated for VMAT, 3D-CRT and step-and-shoot-IMRT (optimized based on Pencil Beam (PB) or Monte Carlo (MC) dose calculation) for typical anal cancer planning target volumes (PTV) including inguinal lymph nodes as usually treated during the first phase (0-36 Gy) of a shrinking field regimen.

**Results:**

With values of 1.33 ± 0.21/1.26 ± 0.05/1.3 ± 0.02 and 1.39 ± 0.09, the CI's for IMRT (PB-Corvus/PB-Hyperion/MC-Hyperion) and VMAT are better than for 3D-CRT with 2.00 ± 0.16. The HI's for the prescribed dose (HI36) for 3D-CRT were 1.06 ± 0.01 and 1.11 ± 0.02 for VMAT, respectively and 1.15 ± 0.02/1.10 ± 0.02/1.11 ± 0.08 for IMRT (PB-Corvus/PB-Hyperion/MC-Hyperion). Mean TTT and MU's for 3D-CRT is 220s/225 ± 11MU and for IMRT (PB-Corvus/PB-Hyperion/MC-Hyperion) is 575s/1260 ± 172MU, 570s/477 ± 84MU and 610s748 ± 193MU while TTT and MU for two-arc-VMAT is 290s/268 ± 19MU.

**Conclusion:**

VMAT provides treatment plans with high conformity and homogeneity equivalent to step-and-shoot-IMRT for this mono-concave treatment volume. Short treatment delivery time and low primary MU are the most important advantages.

## Introduction

**Figure 1:**
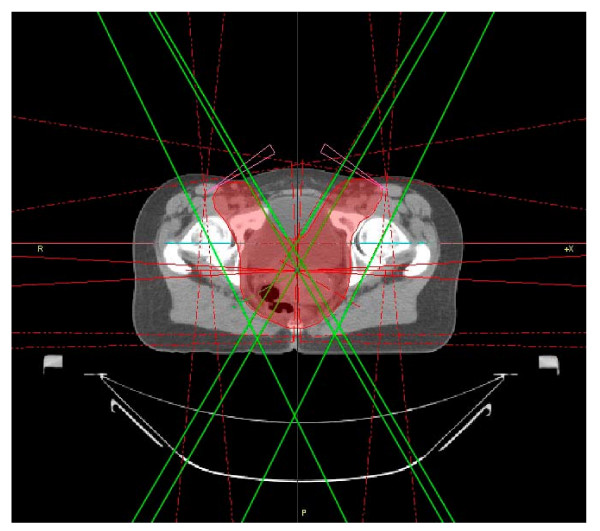
**Axial CT for 3D-CRT with PTV and 6 beams**.

Coverage of large planning target volumes (PTV) as they are treated during the initial part of the protocols for anal cancer is difficult because protection of critical organs is important for the patient's quality of life (QOL) [1]. Until recently, the standard approach has been three dimensional conformal radiotherapy (3D-CRT), typically using a 4-field box technique [2]. The target volume for anal cancer is currently actively being discussed and a consensus document has recently been published by the RTOG [3]. It is, however, not a consequence of specific clinical data but the result of a highly subjective approach (superposition of targets drawn by several individuals) and issues such as vaginal sparing still require cautious evaluation. The PTV therefore still ususally comprises primary tumor and lower external and internal iliac lymph nodes. Medial inguinal lymph nodes are usually treated up to at least 30.6-36 Gy [4,5] and in case of involvement higher doses are required (50.4-54 Gy). Treating inguinal lymph nodes and pelvic lymph nodes simultaneously leads to a mean PTV size of more than 2.750 cm^3 ^as exemplified in figure 1 and such relatively large PTVs are still considered appropriate in recent reviews [6]. Previous studies showed that IMRT provides PTV coverage similar to conventional techniques and at the same time efficiently spares OAR [7]. On the downside, however, IMRT resulted in longer treatment time and a higher number of monitor units (MU). While step-and-shoot IMRT has become more efficient recently [8-10] rotational modulated therapy may be another approach to improve these parameters [11,12]. Volumetric modulated arc therapy (VMAT) is based on the intensity modulated arc therapy (IMAT) paradigm, first described by Yu et. al [13,14]. The basic IMAT idea is to segment on calculated fluences, VMAT on the other hand segments on given structures. Several research groups developed their own IMAT solutions in order to study and exploit its potential for the reduction of treatment time and MU while increasing the number of incident beam directions [15-19], with large target volumes such as encountered with whole abdominopelvic radiotherapy (WAPRT) being particularly in the focus of the group from Ghent [20,21].

Only recently commercial treatment planning systems (TPS) were released for modulated arc therapy. Otto introduced a single-arc VMAT approach [22] that formed the basis for RapidArc^© ^(Varian Medical Systems, USA) that in its first clinical commercial implementation was then evaluated by Cozzi et. al[23] and Palma et. al [16]. ERGO++ (Elekta, Sweden) has been released in parallel as a commercial VMAT system and was evaluated in this study. To provide comprehensive data, VMAT was compared with a complex 3D-CRT technique (6 fields) and step-and-shoot IMRT including Monte Carlo and Pencil Beam calculation. Several strategies (single and dual rotations) were computed, and analysed with regard to dose-volume-histograms (DVH), homogeneity, conformity, exposure of OAR and treatment efficiency (treatment time and monitor units).

## Methods and materials

### Patient anatomy

Eight CT datasets of patients treated at our department for anal cancer were the basis for this study. The PTV was typical for the initial treatment series including the primary tumor, pelvic and inguinal lymph nodes (figure 1). It is to be treated at daily doses of 1.8 Gy to a cumulative dose of 36 Gy. In patients without involved inguinal lymph nodes, the PTV would then be reduced to a typical pelvic PTV without coverage of inguinal lymph nodes. Finally, a boost would be delivered to the primary tumor, its dose depending on tumor size.

Since the initial PTV used in all patients was the most complex one, evaluation of VMAT is only done in this context. Bladder, small intestine, gonads and femoral heads were contoured as OAR. A wedge-shaped anterior auxiliary structure was generated to facilitate the planning process.

### Treatment planning systems

#### 3D-CRT (Masterplan)

3D-CRT-plans were generated with Masterplan 3.1 (Nucletron, The Netherlands). The field geometry consisted of 6 fields as suggested by Götz and Kiricuta [24]. A standard 4 field box treated at an energy of 23 MV and beam angles of 0/87/180/273 degrees was supplemented by 2 oblique auxiliary fields (energy 6 MV) from 30 and 330 degrees, both with 30 degree wedges (figure 1). These additional beams cover the inguinal extensions of the PTV in the anterior/lateral direction. Dose is calculated based on a pencil beam (PB) algorithm.

#### IMRT Treatment Planning

The primary beam setup for the step-and-shoot approach consisted of 9 isotropic nonopposing coplanar beams, both for treatment plans generated with Corvus and Hyperion.

#### IMRT (Pencil Beam, Corvus)

Corvus 6.3 (Best Nomos, USA) is a fully inverse treatment planning system that uses a simulated annealing algorithm for the beamlet optimization process [25]. Dose calculation is based on a PB algorithm.

#### IMRT (Pencil Beam/Monte Carlo, Hyperion)

Hyperion (University of Tuebingen, Germany [26]) has two major innovative features: evidence-based biological modelling and X-ray voxel-based Monte Carlo (XVMC) dose computation including multiple photon transport, electron history repetition and continuous boundary crossing used during optimization and final calculation [27,28]. The system therefore represents several recent advances in IMRT planning. To evaluate the effect of MC dose calculation and optimization we generated plans both based on the PB as well as on the MC algorithm.

### VMAT (ERGO++)

ERGO++ 1.7.1 (3D Line Medical Systems/Elekta) uses a PB algorithm for dose calculation. ERGO++ offers the possibility to adapt the multi-leaf-collimator (MLC) dynamically to the target structure during the rotation. Dose rate, gantry speed and the collimator angle can be modified during the rotation. For our analysis, however, we used a fixed collimator angle since preliminary studies did not suggest an additional gain of optimized collimator angle for the PTV geometry studied. The starting point of the planning/optimization process is the definition of different arrangements of the static control points which divide the arcs into subarcs and the initial manual MLC adaptation to the target volume. The arc modulation optimization algorithm AMOA computes the weighting of each subarc, depending on dose constraints for PTV and each OAR, and consequently defines the dose rate/MU-number for each subarc. Afterwards the sequencer converts the control points into optimized arcs by using predefined rules.

First we analysed different single-rotation paradigms and a dual-rotation approach on the basis of a typical patient/PTV geometry. The single-arc strategies were: **one **360° rotation conforming the collimator to the PTV with shielding of the auxiliary structure when it is in front of the PTV ('**1RotiFo**') and **one **360° rotation on the PTV with full shielding of the auxiliary structure (**'1RotALLW'**).

**Figure 2:**
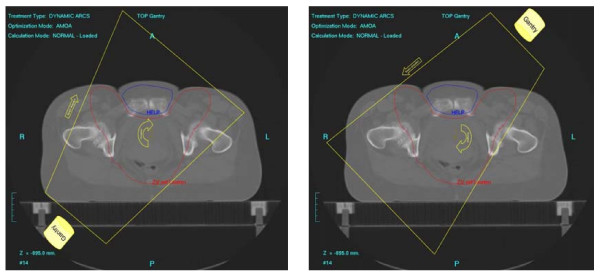
**Two discrete steps during the first rotation without shielding of OAR**.

**Figure 3:**
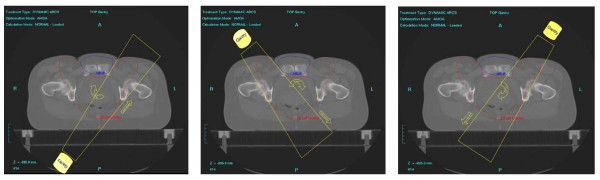
**Three discrete steps during the second rotation with shielding**.

The dual-rotation strategy (**'2Rot'**) used **two **rotations with a starting angle of 181° and a stop angle of 179° each (total of 358°/rotation). These two arcs are subdivided into 72 subarcs for each rotation which results in one control point every 5 degrees. The first rotation treated the whole PTV-horns without sparing any OAR (figure 2). The second rotation around the patient treated the PTV with permanent shielding of the auxiliary structure located between the anterior/lateral PTV-bulges (figure 3) with a margin of 5 mm between the PTV projection and the leaf edges. After this initial evaluation step, the approach with the best overall plan quality (the dual-rotation strategy) was evaluated for all 8 treatment planning CTs.

#### Treatment devices

IMRT, VMAT and 3D-CRT plans were compted for and delivered with an Elekta Synergy^® ^linear accelerator with an energy of 6 MV and a dose rate of 600 MU per minute (MU/min). 3D-CRT, step-and-shoot IMRT plans and VMAT plans were delivered through the MOSAIQ record-and-verify (R&V) system V1.5 (IMPAC Medical Systems Inc./Elekta) with VMAT plans delivered through the most recent release of the console software desktop (V7.0.1).

### Plan comparison

We compared the calculated dose distributions of all four planning systems for sagittal, coronal and lateral planes. The selected patient cases from our database including all contours for OAR and PTV were identical for every planning system. Specifically, DVH parameters such as minimal, mean and maximal dose in the PTV and the OAR's as well as fractional exposure of non-PTV normal tissue was evaluated. Treatment efficiency was quantified by measuring/calculating total treatment time (TTT) and MU (beam-on-time plus time for necessary gantry movements). Finally, we calculated the homogeneity index (HI) and a modified conformity index (CI) which are objective values to describe how well the dose distribution conforms to the shape of a radiosurgical target [29]. The CI was modified to accommodate the fact, that we prescribed dose to the median dose level in the PTV, thus invalidating the classical definition of CI. We therefore defined CI as follows:

(1)

with *V*_D99% _describing the total volume in cm^3 ^which receives the effective minimal target dose (Dose encompassing 99% of the PTV) and *V*_PTV _being the target volume in cm^3^. This definition of CI has the advantage that the value for the minimal dose applied to the target characterizes CI which is in the spirit of the original definition by RTOG. HI is defined according to the RTOG guidelines as follows [30]:

(2)

with *D*_max _being the maximum dose in the treatment plan and *D*_prese _being the prescription dose.

## Results

### Evaluation of different VMAT strategies

**Figure 4:**
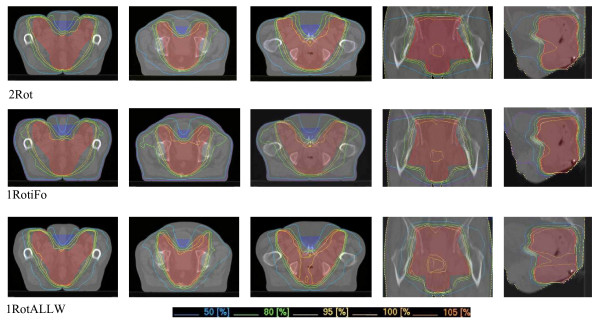
**Dose distributions for different VMAT strategies**. Best homogeneity for 2Rot and best conformity and dose sparing in normal tissue for 1RotALLW.

Figure 4 shows axial, sagittal and coronal dose distributions (DD) for one selected patient generated by the three different VMAT strategies. The DD differ with regard to OAR sparing between the anterior inguinal PTV-extensions, the dose gradient in non-PTV normal tissue, as well as in conformity and homogeneity (figure 4). The '2Rot' strategy provides the best conformity and homogeneity but also the highest dose exposure to the region between the inguinal PTV extensions (maximum of 28.8 Gy) and requires the longest treatment time by using 2 rotations. In contrast, '1RotALLW' creates a steeper dose gradient in the normal tissue encompassed by the PTV and thus better protects the anterior OARs (maximum of only 18 Gy). It also exposes non-PTV tissue to lower integral doses and TTT is significantly shorter. Overall conformity and homogeneity, however, are somewhat inferior due to less modulation during just one rotation. The third strategy, '1RotiFo', represents a mixed solution with intermediate conformity, using only one rotation but providing dose homogeneity similar to what is achieved with the '2Rot' approach, less dose to the OARs but the highest integral dose to non-PTV tissue.

**Figure 5:**
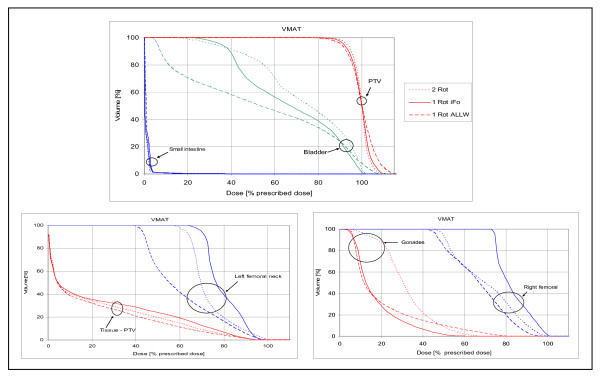
**OAR and PTV DVH's of the VMAT strategies**. The VMAT '2Rot' (dotted line, best homogeneity), VMAT '1RotiFo' (solid line) and VMAT '1RotALLW' (dashed line, best dose sparing in OAR and tissue-PTV).

DVH analysis (figure 5) showed best PTV coverage for '2Rot' with the highest D_99% _and the smallest volume exposed to high doses. '1RotALLW' was inferior regarding PTV coverage and homogeneity while '1RotiFo' showed PTV coverage similar to dual-rotation VMAT.

These differences as parametrized by using CI and HI and in addition the differences in dose exposure to fractional volumes are displayed in table 1.

**Table 1:** DVH parameters for the different VMAT techniques

	**2Rot**	**1Rot iFo**	**1RotALLW**
HI	1.1	1.1	1.15
CI	1.46	1.54	1.49
MU	287	293	348
TT	370s	185s	188s
V_Tissue 10% PD_	13414 cm^3 ^≡ 38.98%	13643 cm^3 ^≡ 39.6%	12456 cm^3 ^≡ 36.2%
V_Tissue 30% PD_	10345 cm^3 ^≡ 30.1%	10635 cm^3 ^≡ 30.9%	8741 cm^3 ^≡ 25.4%
V_Tissue 50% PD_	7571 cm^3 ^≡ 22.0%	8157 cm^3 ^≡ 23.7%	5321 cm^3 ^≡ 15.5%
V_Tissue 70% PD_	4089 cm^3 ^≡ 11.9%	4735 cm^3 ^≡ 13.8%	2727 cm^3 ^≡ 7.9%
V_Tissue 95% PD_	430 cm^3 ^≡ 1.2%	370 cm^3 ^≡ 1.1%	0 cm^3 ^≡ 0%
D_95% Vol PTV_	33.84 Gy ≡ 94%	33.48 Gy ≡ 93%	32.4 Gy ≡ 90%
D_95% Vol Tissue_	0.75 Gy ≡ 2.09%	0.8 Gy ≡ 2.22%	0.02 Gy ≡ 0.06%

Although treatment time for the 2-rotation strategy was almost double that of the single-rotation approaches in this example, further preliminary studies showed that this particular case marked the upper limit of the treatment times and that on average shorter treatment times could be expected also with the 2-rotation approach. Since this technique provided the best conformity and homogeneity we chose it as the benchmark for the following comparison of VMAT with 3D-CRT and fixed beam IMRT.

### Comparison of VMAT and other techniques

**Figure 6:**
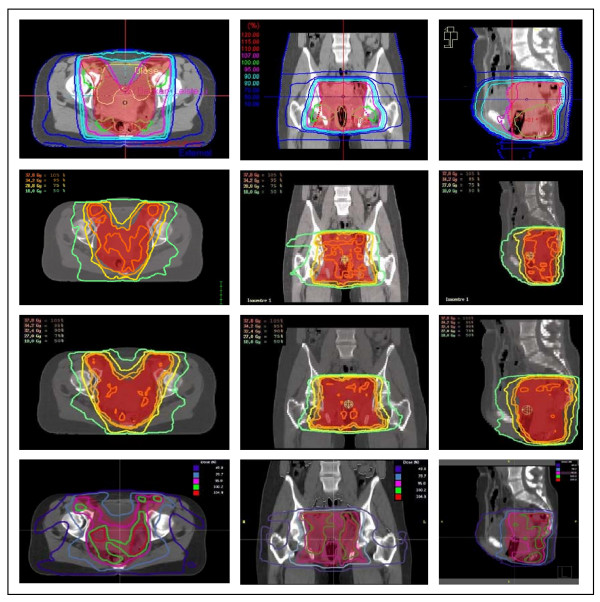
**Axial, coronal and sagittal dose distribution for OTP (3D-CRT), IMRT Hyperion with MC, IMRT Hyperion with PB and Corvus with PB (top to bottom)**.

Figure 6 shows the dose display for all treatment modalities for a typical patient with the PTV delineated in transparent red. The VMAT DD's were already shown in figure 4. For all treatment techniques the IRCU50 prescription guidelines (homogeneity -5% and +7% prescription dose PD) were aimed for but minor deviations had to be accepted as it is usually the case with modulated RT when a realistic treatment plan complexity (number of segments/rotations) for a treatment plan efficiency that is clinically applicable is used. Using our specific treatmtent plan normalization to 50% volume and 50% PD [31], minor compromises were made on the side of both coverage and homogeneity, as reported in table 1. The DD for 3D-CRT shows good homogeneity (no hot or cold spots) but the largest region of non-PTV tissue exposed to high doses. The IMRT Hyperion DD are highly conformal but less homogeneous than 3D-CRT or VMAT "2Rot". Hyperion provides the option to perform PB as well as MC based optimization/calculation. In PB-based calculations, lateral scattering and linear attenuation of x-rays in the patient are not modelled correctly. As consequence, the PB dose distributions look much smoother and subjectively better than MCPB based calculation showing more homogeneous dose distributions than MC. While MC-based plans are more precisely reflecting true absorbed dose, PB was calculated to provide comparison data on the same calculation basis as for the other systems. IMRT Corvus DD has the worst homogeneity and less conformity. Best anterior OAR sparing is performed by IMRT Hyperion.

**Figure 7:**
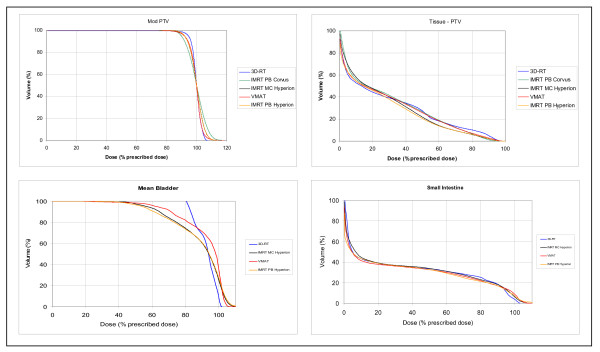
**DVH comparison of VMAT '2 Rot', IMRT and 3D-CRT**.

For DVH generation and comparison (figure 7), all treatment plans were normalized to 36 Gy to the median dose level in the PTV. The highest minimal dose and the lowest maximal dose for the PTV was achieved by 3D-CRT, followed by VMAT "2Rot", IMRT Hyperion and finally IMRT Corvus. The best non-PTV tissue sparing was performed by IMRT Hyperion, the worst by 3D-CRT. Analysing the DVH for bladder, the lowest dose exposure to bladder was acheived by IMRT Hyperion, followed by VMAT "2Rot" and almost no sparing with 3D-CRT. The DVH's for small intestine show no big differenes.

**Figure 8:**
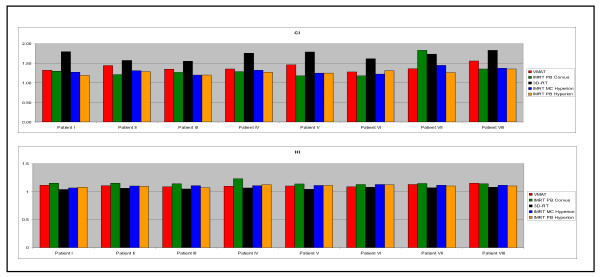
**HI and CI for all individual patients**.

Figure 8 and table 2 indicate the best HI but the worst CI for 3D-CRT. VMAT and IMRT are similar regarding CI and HI, consistently for all individual plans. With values of 1.07 to 1.15 for HI (table 2) all planning systems are within the RTOG recommendations [30].

**Table 2:** Mean TT, MU-number, CI and HI for the three planning systems

	**3D-CRT**	**VMAT '2Rot'**	**IMRT (MC Hyperion)**	**IMRT (PB Hyperion)**	**IMRT (PB Corvus)**
HI	1.06 ± 0.02	1.11 ± 0.02	1.11 ± 0.08	1.10 ± 0.02	1.15 ± 0.03
CI	2.00 ± 0.16	1.39 ± 0.09	1.30 ± 0.02	1.26 ± 0.05	1.33 ± 0.21
MU	225 ± 11	268 ± 19	748 ± 193	477 ± 84	1260 ± 172
TT	220s	290s	610s	570s	575s
V_Tissue 10% PD_	10739 cm^3 ^≡ 48.8%	10463 cm^3 ^≡ 47.6%	10806 cm^3 ^≡ 48.1%	10347 cm^3 ^≡ 46.0%	10591 cm^3 ^≡ 47.5%
V_Tissue 30% PD_	8187 cm^3 ^≡ 37.3%	7674 cm^3 ^≡ 34.9%	7593 cm^3 ^≡ 33.8%	7199 cm^3 ^≡ 32.0%	7874 cm^3 ^≡ 35.3%
V_Tissue 50% PD_	6052 cm^3 ^≡ 27.6%	5089 cm^3 ^≡ 23.1%	4203 cm^3 ^≡ 18.7%	3971 cm^3 ^≡ 17.7%	5186 cm^3 ^≡ 23.2%
V_Tissue 70% PD_	3428 cm^3 ^≡ 15.6%	2734 cm^3 ^≡ 12.4%	1939 cm^3 ^≡ 8.6%	1933 cm^3 ^≡ 8.6%	2612 cm^3 ^≡ 11.7%
V_Tissue 95% PD_	982 cm^3 ^≡ 4.5%	208 cm^3 ^≡ 0.9%	14 cm^3 ^≡ 0.0%	0 cm^3 ^≡ 0.0%	53 cm^3 ^≡ 0.2%
D_95% Vol Tissue_	1.97 Gy ≡ 5.46%	0.75 Gy ≡ 2.09%	0.35 Gy ≡ 0.98%	0.31 Gy ≡ 0.85%	0.52 Gy ≡ 1.45%
D_95% Vol PTV_	34.09 Gy ≡ 94.7%	33.84 Gy ≡ 94%	33.05 Gy ≡ 91.8%	32.95 Gy ≡ 91.54%	32.33 Gy ≡ 89.8%

As parametrized by MU-number and TTT (table 2), 3D-CRT and VMAT "2Rot" are the fastest/most efficient techniques. TTT is 50% shorter than for IMRT and mean MU-number is reduced by more than 70%.

## Discussion

VMAT combines the advantages of conventional 3D-radiotherapy (3D-CRT) with its fast delivery and low number of monitor units (MU) and the advantages of IMRT with the conformal dose distribution (DD) and the reduced dose to critical OAR in when target volumes are irradiated according to recently published recommendations [6].

The benefit of IMRT over 3D-CRT regarding high dose conformity and OAR sparing for pelvic tumors and specifically anal cancer was shown earlier [2,7,32-36]. Chen et al. compared IMRT and 3D-CRT (AP-PA photons with en-face electrons) for anal cancer and they could show that while PTV coverage of IMRT and 3D-CRT were comparable, surrounding OAR received less dose exposure with IMRT [7]. Mundt et al and Roeske et al. analysed whole pelvic radiation for gynecologic malignancies and concluded that IMRT reduces the volume of normal tissue receiving high doses [32] resulting in fewer small bowel complications [35] while retaining PTV coverage. Toxicity and clinical outcome of IMRT for anal cancer was analysed by Milano et al [34]. The group could reduce the radiation dose to normal structures with IMRT and reported a reduction of acute and late toxicities. On the other hand, the increased delivery time allows the repair of sublethal damage (SLD) in tumour cells and might reduce the biological effect [37,38]. Though the relevance of this issue is unclear with TTT having been reduced since the introduction of IMRT and initial reports on dose-protraction effects [39-41], shortening treatment times to ~5 min will completely obviate this discussion.

Since we studied a PTV paradigm with a moderate cranial extension we did not explicitly evaluate bone marrow sparing in the iliac crest, which is in line with the data of Menkarios et al., who had extensively discussed the merit of modulated treatment for anal cancer [2]. They had stressed the technical feasibility and potential benefit of IMRT with regard to bone marrow sparing for PTVs with a high upper limit. Their data, however also shows that for targets with a low upper limit, such as ours, there is no relevant exposure of the iliac crest with any of the studied techniques.

In our evaluation, VMAT, IMRT and 3D-CRT provide almost the same dose coverage in the target but 3D-CRT exposes the surrounding tissue and consequently the OAR to much higher doses. Sparing of bladder and possibly small bowel between the inguinal lymph nodes included in the PTV, however, is not adequately achieved by 3D-CRT.

So far, commercial planning systems for IMAT/VMAT are a not widely spread and initial data were collected with investigational systems, such as those of the groups from Beamount Hospital, Ghent and Vancouver. These initial reports suggested that VMAT may improve the effiency of modulated radiation therapy. Duthoy et al. reported on the feasability of whole abdominopelvic RT using IMAT with a low number of MU's (444 MU) [20] and also reported short treatment times (6.3 minutes) for the treatment of rectal cancer [21].

Both single- and multiple-arc approaches are currently being established clinically for VMAT, showing similar potential for reducing treatment time when plans of equal quality are generated [40]. Clinical implementation of these techniques has also prompted reports on appropriate QA paradigms [41,42].

A single-arc therapy approach was devised by Wang et al The group used a commercial planning system to optimize the intensity profiles of a treatment plan with 36 equi-spaced static beam angles and exported these profiles to an investigational sequencing algorithm to generate a single-arc plan, recalculated with a MC algorithm that was also developed in-house. They investigated multiple target locations and found that their arc-modulation-radiation-therapy (AMRT) paradigm is capable of creating conformal treatment plans, comparable to other IMRT techniques. A reduction of treatment time by ~50% was observed with slightly lower number of MU's for AMRT [42].

Finally, Otto introduced a single arc rotation paradigm increasing treatment efficiency by reducing delivery time to 1.5-3 min which is in the range of what we report in this evaluation. The report was focused on the theoretical basis and technical details of the approach [22] but for a single head-and-neck patient case discussed in his manuscript he reported a treatment time of 107s for VMAT vs. 426s for IMRT with identical dose rate settings.

Palma et al. compared an early prototype of Varian's RapidArc (Varian Medical Systems, Palo Alto, CA) technique with 3D-CRT and fixed field dynamic IMRT for prostate cancer. On a predominantly spherical target, they reported, similar to our results, a higher treatment efficiency for VMAT (491 MU constant dose rate/454 MU variable dose rate) vs. 789 with IMRT as well shorter treatment times [16], though the absolute level of MU was higher in their series than in our comparison, reflecting an earlier development stage of both modalities. IMRT and VMAT provided better dose distributions than 3D-CRT. A comparison of non-PTV tissue was not performed and can therefore not be assessed.

The most recent report was provided by Cozzi et al. using an improved version of the RapidArc prototype but with focus on a larger PTV (cervix uteri). They reported a similar PTV coverage of VMAT (single rotation, variable dose rate: up to 600 MU/Min) and IMRT (sliding window, 5 beams, fixed dose rate: 300 MU/min) with improved homogeneity, better conformity and a major reduction of OAR irradiation. Our results showed identical homogeneity for IMRT and VMAT but higher conformity of the IMRT approach. Although a detailed comparative analysis of the two series is not possible, this difference is most likely a consequence of the higher number of incident beams - and possibly more modulation - used in our comparison. The different geometry of the PTV might also factor in (PTV encompassing pelvic nodes only in their series vs. pelvic and inguinal nodes in ours). Cozzi et al. reported VMAT delivery with less than 2 min delivery time and less than 245 MU/fraction. A direct comparison of IMRT and VMAT to our situation was not possible due to the fact that we used a step-and-shoot IMRT with 9 beams and a dose rate of 600 MU/min.

While the relative merit of the different modulation approaches with regard to PTV coverage and OAR sparing cannot finally be assessed, the constant reduction in treatment time and MU used for all approaches has now reached a level at which any further discussion about detrimental effects of treatment protraction [37,38,41] or secondary tumors due to the higher primary number of MU necessary for modulated therapy [22,43] is futile.

## Conclusion

VMAT is an efficient treatment modality for large and moderately complex pelvic targets already in its initial developmental implementation. While in this situation dose homogeneity and high dose conformity approach that of highly modulated fixed beam IMRT, treatment times and MU are further reduced. Further investigations will show how efficient VMAT can handle other target volumes and evaluate the delivery accuracy of this complex treatment technique with multiple dynamical changes during rotation.

## Competing interests

The authors declare that they have no competing interests.

## Authors' contributions

FS conceived the experiment design, carried out the experimental work of the study and drafted the manuscript. DW participated in conceiving the study and helped to draft the manuscript. VS, FLo and YAM have been involved in data interpretation. FL and FW have been involved in data interpretation and drafting the manuscript. SM participated in conceiving the study and helped drafting the manuscript. All authors read and approved the final manuscript.
